# Management and Prognostic Factors for Thyroid Carcinoma Showing Thymus-Like Elements (CASTLE): A Case Series Study

**DOI:** 10.3389/fonc.2018.00477

**Published:** 2018-10-26

**Authors:** Rui Gao, Xi Jia, Ting Ji, Jinteng Feng, Aimin Yang, Guangjian Zhang

**Affiliations:** ^1^Department of Nuclear Medicine, The First Affiliated Hospital of Xi'an Jiaotong University, Xi'an, China; ^2^Department of Thoracic Surgery, The First Affiliated Hospital of Xi'an Jiaotong University, Xi'an, China

**Keywords:** thyroid tumor, CASTLE, surgery, radiotherapy, extrathyroidal tumor extension, lymph node metastasis

## Abstract

**Introduction:** This study aims to identify the prognostic factors that influence therapeutic modalities for thyroid carcinoma showing thymus-like elements (CASTLE).

**Materials and Methods:** Reported studies with CASTLE patients published between 2004 and 2018 were retrieved from a cross-database literature search. Three patients treated in our institute were also included as one case series. Standardized data collection was performed; data pertaining to clinical stages, treatment regimens, and survival time were collected and statistically analyzed.

**Results:** Up to 26 case series of CASTLE were selected, including 51 males and 38 females with a median age of 48 years. Follow-up time ranged from 2 to 362 months and the median survival time was 158.03 months. Lymph node metastasis and tumor invasion of adjacent tissue both showed a significant negative effect on survival (*p* = 0.001 and 0.013, respectively). Radiotherapy significantly improved survival (*p* = 0.034), while neck dissection significantly prolonged survival only in patients with extrathyroidal extension (*p* = 0.043).

**Conclusions:** Extrathyroidal infiltration and nodal metastasis are important factors in cancer outcomes. Radiation therapy appears to be important for better outcomes in CASTLE patients, and neck dissection is recommended for patients with extrathyroidal extension.

## Introduction

Carcinoma showing thymus-like elements (CASTLE) is a rare intrathyroidal malignant neoplasm and is thought to arise from ectopic thymus or branchial pouch remnants. It was named by Chan and Rosai in 1991 and was designated as an independent clinicopathologic thyroid tumor entity by the World Health Organization in 2004 (1, 2). To date, approximately 300 cases of CASTLE have been reported in the literature (3). Clinical features, imaging findings, and even fine needle aspiration biopsy (FNAB) manifestations of CASTLE are commonly found in other advanced thyroid malignancies; therefore, its definitive diagnosis often relies on pathological findings, particularly immunohistochemistry studies postsurgery (3–5). Under such circumstances, the surgical choice is an empirical rather than evidence-based decision.

The prognosis for patients with CASTLE is relatively favorable; however, there have also been aggressive cases reported with a high likelihood of early recurrence and metastasis (6–9). The choice of appropriate treatment strategy, including surgical procedure and postoperative therapeutic regimen, is imperative for a good prognosis for these patients. However, due to the rarity of this disease, no large cohort study aiming to find a definitive treatment strategy has been reported. In this study, we report three clinical cases of CASTLE, along with a review of reported cases, attempting to define the prognostic factors that may influence the treatment plan and to discuss different therapeutic modalities for CASTLE. To the best of our knowledge, this is the first study of its kind.

## Materials and methods

### Literature retrieval and data extraction

**Figure 1:**
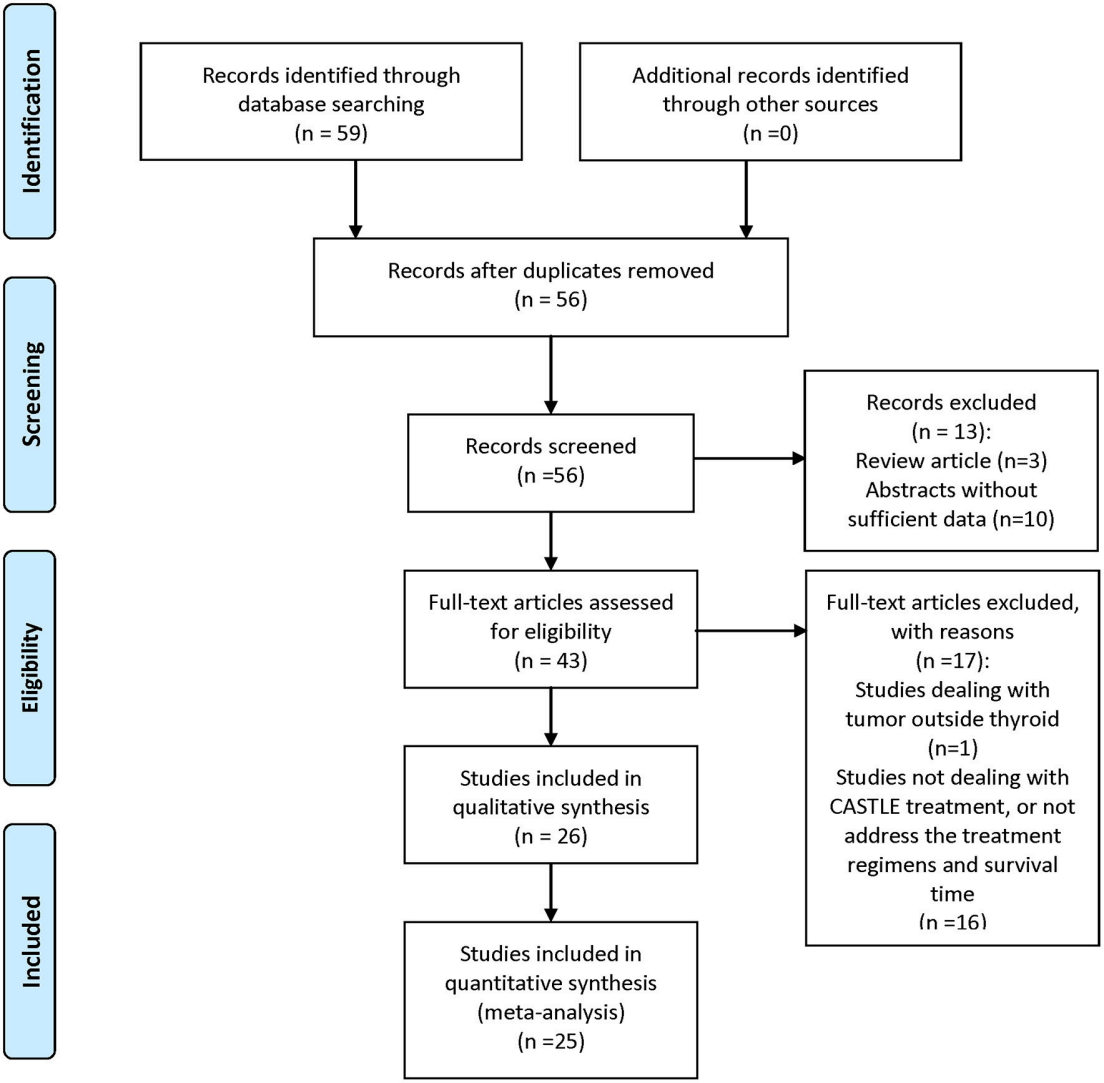
Selection algorithm of retrieved literatures.

A comprehensive search of PubMed, EMBASE, Cochrane Library, Science Citation Index, and Web of Science was performed. Keywords used were “CASTLE,” “thyroid,” “carcinoma,” “thymic,” and all their combinations. Additional studies were identified by using the “related articles” feature in PubMed. Where more than one publication of a single trial existed, only the publication with the most complete data was included. Each retrieved article was assessed carefully for the following inclusion criteria: (1) CASTLE was histologically or cytologically confirmed; (2) clinical features and treatment regimen pertaining to each patient were reported; and (3) overall survival time of the patients was given. The literature review process is illustrated in Figure 1. Patients in each of the included studies were considered as one case series.

Three CASTLE cases treated at The First Affiliated Hospital of Xi'an Jiaotong University were also included as one case series. The diagnosis of all three cases was verified using histologic examination and immunohistochemical positive staining of CD5, and negative staining of thyroglobulin and thyroid transcription factor-1 (TTF-1). Survival time was defined as the time between treatment and death. Data pertaining to clinical features of CASTLE, treatment regimens, follow-up time, and survival status at final follow-up evaluation of all the case series were collected. Individual patient data analysis was conducted using methods described and validated in previous studies (10, 11). Briefly, the individual patient data meta-analysis statistical approach pools all published raw patient data as if all patients belonged to a single cohort to perform a survival analysis, instead of basing calculations on summary statistics as in traditional meta-analysis methodology. Studies were analyzed by institution and author to ensure that patients were not duplicated.

This study was carried out in accordance with the recommendations of the Ethics committee of The First Affiliated Hospital of Xi'an Jiaotong University, and with written informed consent in accordance with the Declaration of Helsinki. Written informed consent was obtained from all the participants for the publication of this case report.

### Statistical analysis

For comparison of median survival time (MST) based on different treatment regimens, we pooled the events from the cases in each group as if they came from a single study (10, 11). The Kaplan–Meier method was used to obtain the survival curves, and the log-rank test was employed to determine the MST differences between groups. The chi-square test was used to compare treatment modalities between the different groups (with/without nodal involvement/extrathyroidal extension). *p* < 0.05 was considered statistically significant. SPSS 13.0 software (SPSS Inc., Chicago, IL, USA) was used for statistical analysis.

## Results

### The three castle patients in our institute

Three patients with CASTLE were diagnosed and treated at The First Affiliated Hospital of Xi'an Jiaotong University between January 2008 and December 2018. Clinicopathological data, therapeutic procedures performed, and clinical outcomes were elucidated for each patient as follows.

#### Patient 1

Patient 1 was a 61-year-old female who presented with a 5-cm anterior neck mass with retrosternal extension. She was surgically treated with a right thyroid lobectomy at another institution. The lesion was interpreted to be a poorly differentiated papillary thyroid cancer. Postoperative treatment consisted of sequential and combined chemoradiotherapy. Local recurrence in the region of the right thyroid occurred 20 months later, and radiotherapy was performed to relieve symptoms. Six years later, the patient had local recurrence again and was referred to our institution for further treatment. After curative wide local excision and central neck dissection, postoperative radiotherapy was given to the thyroid bed and bilateral cervical lymph node areas (55 Gy/16 fractions). Histological re-evaluation of the recurrent tumor specimens revealed CASTLE (pT4bN1M0). A third local recurrence was diagnosed 1 year later, and concurrent chemotherapy (paclitaxel 210 mg/m^2^ and cisplatin 40 mg/m^2^) and radiotherapy (50.4 Gy /28 fractions) were applied. The patient died from acute respiratory distress 1 month after the initiation of treatment.

#### Patient 2

Patient 2 was a 48-year-old male pathologically diagnosed with stage pT4aN0M0 CASTLE postsurgery. He presented with rapidly growing thyroid nodules with fixation to underlying structures. He underwent total thyroidectomy with central neck dissection. On exploration, the thyroid mass was seen extended to the left laryngeal nerve, strap muscle, trachea, and esophagus. Curative wide local excision was attempted on patients with gross residual local disease. Postoperative external beam radiation therapy (50.4 Gy/28 fractions) was given to the thyroid bed and bilateral cervical lymph node area. He remained well 2 years post therapy with no palpable neck disease.

#### Patient 3

Patient 3 was a 67-year-old female who presented with hoarseness and dysphagia for a few weeks. CT scans showed a 3.8 cm left thyroid tumor with superior mediastinal and tracheal involvements, i.e., stage pT4bN0M0. Complete resection of the tumor was successfully achieved by total thyroidectomy and central neck dissection. Postsurgical chemotherapy was applied (docetaxel 120 mg/m^2^ and cisplatin 30 mg/m^2^), followed by a course of intensity modulated radiotherapy (70 Gy at 2.5 Gy per fraction). She remains well 4 years after diagnosis.

### The retrieved case series

Out of the total of 58 reports on CASTLE we found in the databases, 25 were eligible for inclusion. A total number of 88 cases were identified, which, in addition to the above mentioned three patients from our institute, totaled 91 CASTLE patients. Two were excluded because there were no detailed follow-up data, resulting in 89 cases for further analysis. The median age for the occurrence of CASTLE was 48 years (25–76 years, *n* = 89). The disease was more frequently diagnosed in females (51 Female: 38 Male). Furthermore, in 57.14% of described cases, the tumor was found in the lower part of the thyroid (48/84 cases), with no predominance of side (40 left: 39 right: 5 both: 5 unknown).

All the cases, except one, underwent surgery, with a large variation among procedures. The most commonly adopted surgery was lobectomy (46.59%, 41/88 cases). Other types included total thyroidectomy, subtotal thyroidectomy, and palliative surgery. In 68.18% of cases (60/88), patients underwent cervical lymph node dissection (ND). Central compartment (level VI) dissection on the side of the resected thyroid was performed in all cases received ND, while lateral compartment (levels II–V) dissection to various extents was applied in 46.67% (28/60) of cases for possibly existed nodal disease.

More than half of the cases that described nodal status reported lymph node involvement (51.61%, 32/62). The majority of lymph nodes, 84.37% of them, were reported to be from the central compartment (27/32) and 15.63%, from the lateral compartment. A high proportion of our patients (79.69%, 51/64 cases) reported positive extrathyroidal extension of the tumor, mostly to the nearby ipsilateral strap muscles, trachea, and recurrent laryngeal nerves. Furthermore, seven out of the 45 patients with described distant metastasis had lung lesions. The range of the follow-up time was large, varying between 2 and 362 months. Detailed information on all the case series is summarized in Table 1.

**Table 1:** Characteristics of the 25 retrieved case series.

**Author**	**Sample size (*n*)**	**M/F ratio**	**Median age (years)**	**Treatment**	**Extrathyroidal extension**	**Lymph node metastasis**	**Follow up (months)**
(3)	1	1:0	60	TT	Y	1 N	36
(6)	6	2:4	64	3 TT, 2 LT, 1 NA	4 Y, 1 N, 1 UN	3 Y, 2 N, 1 UN	66.5
(7)	1	1:0	46	TT	Y	1 Y	22
(8)	3	2:1	49	1 TT, 2 PT	3 Y	3 UN	30
(12)	2	1:1	39.5	2 TT	2 Y	2 Y	63.5
(13)	3	0:3	51	2 LT, 1 LT^*^	1 Y, 1 N, 1 UN	2 N, 1 UN	206
(14)	2	1:1	65	1 TT^*^, 1 ST	2 Y	1 Y, 1 UN	15
(15)	1	1:0	59	ST	Y	1 UN	17
(16)	1	0:1	47	PT	Y	1 UN	17
(17)	1	0:1	67	LT^*^	Y	1 UN	12
(18)	7	1:6	47	3 LT, 2 ST, 2ST^*^	2 Y, 1 N, 4 UN	2Y, 3 N, 2 UN	132
(19)	1	0:1	41	TT	Y	1 N	12
(20)	1	0:1	54	LT	N	1 UN	36
(21)	4	1:3	60	2 TT, 1TT^*^, 1 ST	3 Y, 1 N	3 Y, 1 N	16
(22)	8	4:4	48.5	1 TT, 1TT^*^, 4 LT^*^, 2 LT	4 Y, 1 N, 3 UN	4 Y, 4 N	12
(23)	1	0:1	41	TT	UN	1 UN	12
(24)	1	1:0	34	LT	UN	1 UN	20
(25)	3	1:2	37	2 LT, 1 TT	1 Y, 2 UN	1Y, 2 UN	20
(26)	7	4:3	51	7 LT	4 Y, 3 N	1 Y, 6 N	34
(27)	6	3:3	53	6 TT	5 Y, 1 N	2 Y, 4 N	32
(28)	1	1:0	26	TT	Y	1 Y	3
(29)	1	0:1	52	TT^*^	Y	1 UN	5
(30)	2	0:2	33.5	LT	UN	1 UN	6.5
(31)	10	4:6	46	10 LT	UN	2 Y, 8 N	38
(32)	14	8:6	48	2TT, 7LT, 1ST, 4PT	9 Y, 3 N, 2 UN	7 Y, 6 N, 1 UN	42

*M, male; F, female; TT, total thyroidectomy; LT, lobectomy; PT, partial thyroidectomy; ST, subtotal thyroidectomy; UN, undefined; NA, not applied; Y, positive; N, negative*.

* *Cases also received perithyroidal tissue resection*.

### MST in patients with different metastatic conditions

**Figure 2:**
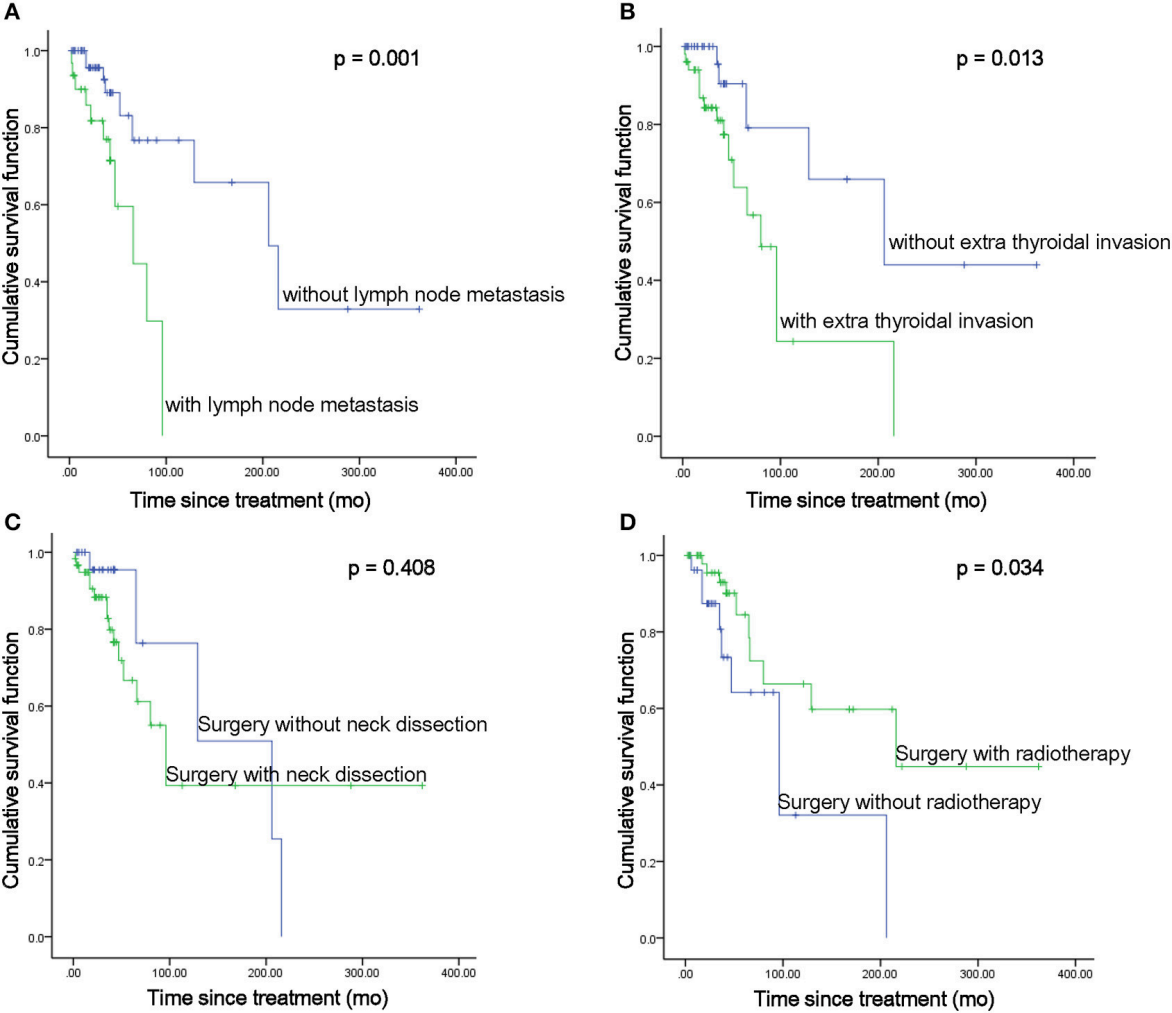
MST comparisons between patients with different disease status **(A,B)** and treatment modalities **(C,D)**.

There were 89 CASTLE patients in this study with an MST of 158.03 months. Among the cohort of patients, data pertaining to lymph node metastatic status were available in 62 CASTLE patients, with 32 of them having nodal involvement. The surgical modalities between patients with/without lymph node metastasis were significantly different (χ^2^ = 8.925, *p* = 0.03). A significantly higher proportion of cases with nodal disease received a total thyroidectomy compared with cases without. The proportion of cases receiving neck dissection, radiotherapy, and/or chemotherapy was not significantly different between the groups of patients with/without nodal disease (Table 2). Patients with lymph node metastasis had significantly shorter survival times compared with cases without nodal involvement (MST 61.89 vs. 212.88 months, respectively, *p* = 0.001, Figure 2A).

**Table 2:** Treatment in the CASTLE patients with advanced status.

	**Tumor extension (*****n*** = **64)**				**Nodal metastasis (*****n*** = **62)**			
	**Positive (*****n*** = **51, 79.69%)**		**Negative (*****n*** = **13, 20.31%)**		**Positive (*****n*** = **32, 51.61%)**		**Negative** (***n*** = **30, 48.39%)**	
Thyroidectomy	***n***	**%**	***n***	**%**	***n***	**%**	***n***	**%**
Total	22	43.14	3	23.08	19	59.38	9	30^*^
Subtotal	5	9.80	1	7.69	3	9.38	2	6.67
Lobectomy	19	37.25	6	46.15	10	31.25	15	50
Palliative	5	9.80	2	15.38	0	0	4	13.33
Neck dissection	43	84.31	10	76.92	31	96.88	29	96.67
Central compartment	17	39.54	5	38.46	13	41.94	10	34.48
Lateral compartment	23	53.49	4	30.77	18	58.06	10	34.48
Radiotherapy	36	70.59	8	61.54	24	75	19	63.33
Combined chemotherapy	11	21.59	0	0	5	15.63	2	6.67

* *p < 0.05 in comparison of the thyroidectomy types between groups of cases with/without lymph node metastasis*.

As expected, the MST of the 51 patients with positive extrathyroidal infiltration was significantly shorter than that of patients with negative extrathyroidal extension (MST 98.14 vs. 232.22 months, respectively, *p* = 0.013, Figure 2B). No significant difference in treatments was discovered between cases with/without extrathyroidal extensions. Not surprisingly, the MST of the seven patients with evidence of lung metastases was significantly shorter as compared with that of the patients with no distant metastatic disease (MST 52.86 vs. 183.61 months, respectively, *p* = 0.001).

### MST in patients treated with different treatment modalities

The surgical approaches taken with these patients fell into two categories: thyroidectomy only, referring to thyroid dissections to different extents (*n* = 28), or thyroidectomy with ND, including therapeutic and prophylactic ND (*n* = 60). ND did not show a significant survival benefit compared with patients who underwent thyroidectomy only (MST 176.66 vs. 153.44 months, respectively, *p* = 0.408, Figure 2C). However, when we focused on the impact of ND in patients with extrathyroidal invasion, it significantly enhanced the survival of the patients with extrathyroidal extension (MST 191.19 vs. 67.93 months, respectively, *p* = 0.043). Though a beneficial effect of ND was also seen in patients without extrathyroidal invasion, no statistical significance was reached (MST 304.96 vs. 133.33 months, respectively, *p* = 0.367). As almost all the cases reported nodal status received ND (Table 2), no statistical analysis was performed to compare the impact of ND in cases with/without nodal disease.

Of the 89 CASTLE patients, 58 were operated on followed by radiotherapy treatment, while the other 31 patients received surgery as the only treatment. The pooled estimates for the two treatment categories showed that radiotherapy significantly improved patient survival (MST 212.48 vs. 106.03 months, respectively, *p* = 0.034, Figure 2D). Further analysis focusing on the impact of radiotherapy on patients with different nodal statuses showed that it significantly enhanced the survival of patients without lymph node metastasis (MST 242.93 vs. 96.45 months, *p* = 0.035). No significant beneficial effect was demonstrated in patients with nodal involvement (MST 112.41 vs. 56.21 months, *p* = 0.102). It did not significantly elongate the survival of the patients with/without thyroidal extensions (MST 138.12 vs. 74.28 months, *p* = 0.241; 279.67 vs. 113.23 months, respectively, *p* = 0.105).

Chemotherapy was usually applied in advanced local disease, i.e., cases with locally widespread disease or with gross residual disease post-surgery. Therefore, a comparison of MST was performed between thyroidal extension-positive cases those who received chemotherapy (*n* = 11) or not (*n* = 40). The MST of the cases that received chemotherapy was significantly shorter than that of the extrathyroidal extension-positive cases that did not receive chemotherapy (MST 45.49 vs. 120.87 months, respectively, *p* = 0.007).

## Discussion

CASTLE is a rare malignant thyroid neoplasm. Reports of this disease have been increasing in recent years, although the total number of reported cases is still less than 300 (1–5). This is likely because the clinical manifestations of CASTLE vary and are not specific to the disease (Table 3), and a conclusive diagnosis mainly relies on postsurgical pathological examination, particularly immunohistochemistry studies.

**Table 3:** Comparison between CASTLE, differentiated thyroid cancer, and anaplastic thyroid cancer.

	**CASTLE (3, 6–8, 12–32)**	**Differentiated thyroid cancer (33–36)**	**Anaplastic thyroid cancer (37–40)**
Morbidity	0.1–0.15% of all thyroid cancer	Over 95% of all thyroid cancer	2–5% of all thyroid cancer
Tumor origin	Thyroid solid cell nests	Thyroid follicular epithelial cells	Thyroid follicular epithelial cells
Susceptible age	50s	20–50 years	55–69 years
Sex	No gender predominance	Women representing about 1/4 of the patients	Women representing 55–77% of all patients
First symptom	Mostly neck mass	Mostly neck mass	Neck pain, dysphagia, hoarseness, stridor, and dyspnea due to the rapidly expanding tumor
Lab tests	Mostly normal	Mostly normal	Mostly normal
Location	Mostly lower pole	No predominance	No predominance
Progression	Mostly indolent	Mostly indolent	Often aggressive
**PATHOLOGY STUDIES**			
FNAC	Sensitivity was only 8.3%	/	/
Gross pathology	Lobulated	Nodular	Tumor surface usually reveals a white- to tan-colored firm surface speckled with necrosis.
Immuno-histopathology	✓ Mostly CD5 positive ✓ May be positive for CD117, p63 et al. ✓ Negative for Tg	✓ Positive for Tg ✓ Mostly positive for TPO, CD57, CK19, galectin3, HBME1	✓ Mostly CK and TP53 positive ✓ Negative for Tg, TTF1, and CEA
**IMAGE TESTS**			
Ultrasound	✓ Lobulated, solid, hypoechoic ✓ Heterogeneous internal echoes ✓ Without cystic components or calcification	✓ Cystic necrosis and calcification ✓ Avid enhancement	✓ Cystic necrosis and calcification ✓ Avid enhancement
CT	✓ A well-defined, soft tissue density mass without calcification ✓ Mostly heterogeneously enhanced	✓ Low or soft tissue density with cystic necrosis and/or calcification ✓ Avid enhancement	✓ Large, solid, and ill-defined masses ✓ Frequently with necrosis, nodular calcification ✓ Direct invasion into adjacent organs, and/or ✓ Lymph node metastasis
MR	✓ Homogeneous isointensity on T1 slightly hyper intense on T2	/	/
SPECT	Cold nodule	Cold nodule	Cold nodule
^18^F-FDG PET/CT	Increased uptake in the thyroid lesion/metastatic lymph nodes	✓ Positive uptake varied from 2.2 to 3.8% ✓ More than half showed a focal uptake pattern	Increased uptake in the thyroid lesion/metastatic lesions
Capsule invasion	About 50–60%	About 6–13%	Over 90%
Tumor extension	About 38%	About 5.7–7%	
Lymph node metastasis	About one-third to 50%	About 35–50%	
**TREATMENT**			
Surgery	First choice	First choice	First choice
Chemotherapy	Should be attempted in patients with advanced or metastatic disease	Not suggested	Chemo radiotherapy was suggested to be performed after surgery
Radiotherapy	Postoperative radiotherapy is considered for patients with positive nodal status	Radioiodine ablation	
Distant metastasis	14–29%	30%	20 to 50%
Prognosis	5- and 10-year CSS rates were 90 and 82%	5- and 10-year CSS rates were 98 and 96%	5-year CSS rates were 5 and 15%

Overall, imaging results, including those obtained from ultrasound, CT, and MRI scans of CASTLE are not specific and are commonly seen in other aggressive head and neck tumors, particularly in thyroid cancer (5). It is worth noting that the rare presence of calcifications could not rule out the diagnosis of CASTLE, as some CASTLE patients have been reported to have calcifications seen in their scans (27). Moreover, CASTLE was described as a “cold nodule” on SPECT (Single-Photon Emission Computed Tomography) imaged with technetium, which is a common manifestation of thyroid malignancies (9). Fluorine-18-fluorodeoxyglucose (^18^F-FDG) positron emission tomography/X-ray computed tomography (PET/CT) may shed some light on this issue, as significantly increased glucose metabolism has been noted in this disease (7, 28). Altogether, FDG PET/CT was used in three cases to make the CASTLE diagnosis and demonstrated obvious focal FDG tumor uptake. This was vastly different from differentiated carcinoma of the thyroid, as it barely shows increased uptake of FDG in the nodule (41, 42). This warrants further studies on the application of FDG PET/CT in the differential diagnosis of these two diseases.

FNAB cytological examinations offer limited diagnostic value for CASTLE (9, 29). Reported cytological differential diagnoses vary, with most cases being identified as anaplastic carcinoma or poorly differentiated carcinoma, without further definite classification [(12–32), Table 3]. CASTLE was correctly identified as malignant in 265 of 269 reported cases using FNAB results, while only in four cases among them, a preoperative diagnosis of CASTLE was given (29). Recent advances in immunophenotypic analysis, including flow cytometry and immunohistochemical analysis, may improve the accuracy of FNAB for diagnosing this thyroid neoplasm.

Disease infiltration to adjacent structures occurred in 79.69% of the included cases and 68.8% of them revealed metastatic disease in regional lymph nodes. The incidence was higher than in previous reports (6, 9), and the inclusion of recently reported local progressive tumors might contribute to the high incidence (7, 26, 32). The high risk of tumor invasion indicates that CASTLE tumors may pursue a more aggressive course oncologically (6–9). More active surgical procedures, i.e., total thyroidectomies, were applied in cases with lymph node metastasis; however, the MST of cases with positive nodes was significantly shorter than that in patients without, indicating that nodal metastasis is a risk factor that drastically affects survival (9, 26, 27). Expectedly, extrathyroidal extension was also shown to be a prognostic factor that significantly influenced the survival of patients.

Preoperative examinations are usually unable to provide a conclusive diagnosis; therefore, surgery is the primary treatment for CASTLE (11–23). Previous reports supported the need to perform curative surgery with ND to reach favorable outcomes (9, 26, 27). Thus, 68.18% of the reported cases underwent therapeutic ND to remove suspected node disease suggested preoperatively (7 out of 60 cases), or prophylactic ND to eliminate the possibility of metastatic lymph nodes. Though no significant benefit to the survival was found in the pooled data, subgroup analysis showed that ND significantly prolonged the survival of patients with extrathyroidal extensions. Because CASTLE with extrathyroidal extension might be more susceptive to lymph node metastasis, prophylactic ND may be an effective way to lower the incidence of local recurrence. Therefore, thyroidectomy with ND should always be performed, including prophylactic ND in CASTLE patients with clinical N0 neck.

Radiotherapy is commonly used in advanced CASTLE to reduce the regional recurrence rate; however, because of the rarity of CASTLE, data on the efficacy of adjuvant radiotherapy are anecdotal. Our analysis of the pooled data showed that surgery plus radiotherapy significantly improved the survival of CASTLE patients. Interestingly, the beneficial effect was most prominently seen in the cases without lymph node metastasis, suggesting that the application of radiotherapy may not be limited to cases with evidence of metastasis.

There were many different reported chemotherapy agents that had been tried in CASTLE. There were 21.57% of cases with extrathyroidal extensions that received chemotherapy; however, the pooled data failed to provide support for using chemotherapy to improve patient survival. The widespread nature of the disease in cases that required chemotherapy may have contributed to the short survival.

It should be noted that a cohort of 89 cases is still comparatively small and cannot provide a complete picture of the various treatments for this malignancy. The limitations of our study include the potential selection bias that may be present in any retrospective study that uses heterogeneous data. As we mentioned previously, a variety of surgeries were performed on these patients. Though more than half of the cases demonstrated tumor infiltration to extrathyroidal tissue or metastasis to lymph nodes, the proportion of cases that received total thyroidectomy was only about 40%. Lobectomy is still preferred by surgeons when the tumor involves the unilateral thyroid gland. Furthermore, because few metastatic lymph nodes could be documented presurgical, the choice of levels II–V lateral compartment dissection was an empirical decision. The differences in the performance status of patients at diagnosis can also influence the subsequent choice of treatment as well as the prognosis. Therefore, in this circumstance, the comparison of the survival duration between CASTLE patients with different treatment modalities should be interpreted with caution.

In conclusion, thyroid CASTLE is a rare malignancy with various manifestations that are difficult to diagnose prior to surgery. Extrathyroidal extension and nodal metastasis are significant prognostic factors of CASTLE. Lymphadenectomy and radiotherapy should be applied to improve the survival of CASTLE patients with different disease statuses. A cohort of 89 cases is not large enough for a comprehensive understanding of the disease; therefore, the results obtained here need to be updated using further analyses that include more CASTLE case series.

## Author contributions

RG and GZ designed the study and composed the manuscript. TJ provided figures and tables. XJ, JF, and AY had the acquisition, analysis, or interpretation of data for the work.

### Conflict of interest statement

The authors declare that the research was conducted in the absence of any commercial or financial relationships that could be construed as a potential conflict of interest.
